# Coexisiting type 1 diabetes and celiac disease is associated with lower Hba1c when compared to type 1 diabetes alone: data from the Australasian Diabetes Data Network (ADDN) registry

**DOI:** 10.1007/s00592-023-02113-z

**Published:** 2023-06-20

**Authors:** Steven James, Lin Perry, Julia Lowe, Kim C. Donaghue, Anna Pham-Short, Maria E. Craig, the ADDN Study Group, Geoff Ambler, Kym Anderson, Sof Andrikopoulos, Jenny Batch, Justin Brown, Fergus Cameron, Peter G. Colman, Louise Conwell, Andrew Cotterill, Jennifer Couper, Elizabeth Davis, Martin de Bock, Jan Fairchild, Gerry Fegan, Spiros Fourlanos, Sarah Glastras, Peter Goss, Leonie Gray, Peter Shane Hamblin, Paul Hofman, Dianne Jane Holmes-Walker, Tony Huynh, Sonia Isaacs, Craig Jefferies, Stephanie Johnson, Tim Jones, Jeff Kao, Bruce R. King, Antony Lafferty, Michelle Martin, Robert McCrossin, Kris Neville, Mark Pascoe, Ryan Paul, Alexia Peña, Liza Phillips, Darrell Price, Christine Rodda, David Simmons, Richard Sinnott, Carmel Smart, Monique Stone, Steve Stranks, Elaine Tham, Barbara Waddell, Glenn Ward, Ben Wheeler, Helen Woodhead, Anthony Zimmermann

**Affiliations:** 1 University of the Sunshine Coast, Moreton Bay Campus, 1 Moreton Parade, Petrie, 4502 Australia; 2 University of Melbourne, Parkville, Australia; 3 University of Western Sydney, Campbelltown, Australia; 4 University of Technology Sydney, Ultimo, Australia; 5 Prince of Wales Hospital, Randwick, Australia; 6 University of Toronto, Toronto, Canada; 7 Children’s Hospital at Westmead, Westmead, Australia; 8 University of Sydney, Camperdown, Australia; 9 University of New South Wales, Kensington, Australia

**Keywords:** Adolescent, Celiac disease, HbA1c, Insulin pump therapy, Type 1 diabetes, Young adult

## Abstract

**Aim:**

To compare HbA1c and clinical outcomes in adolescents and young adults with type 1 diabetes (T1D), with or without celiac disease (CD).

**Methods:**

Longitudinal data were extracted from ADDN, a prospective clinical diabetes registry. Inclusion criteria were T1D (with or without CD), ≥ 1 HbA1c measurement, age 16–25 years and diabetes duration ≥ 1 year at last measurement. Multivariable Generalised Estimated Equation models were used for longitudinal analysis of variables associated with HbA1c.

**Results:**

Across all measurements, those with coexisting T1D and CD had lower HbA1c when compared to those with T1D alone (8.5 ± 1.5% (69.4 ± 16.8 mmol/mol) vs. 8.7 ± 1.8% (71.4 ± 19.8 mmol/mol); *p* < 0.001); lower HbA1c was associated with shorter diabetes duration (B = − 0.06; 95% CI − 0.07 to − 0.05; *p* < 0.001), male sex (B = − 0.24; − 0.36 to − 0.11; *p* < 0.001), insulin pump therapy use (B = − 0.46; − 0.58 to − 0.34; *p* < 0.001), coexistence of T1D and CD (B = − 0.28; − 0.48 to − 0.07; *p* = 0.01), blood pressure (B = − 0.16; − 0.23 to − 0.09; *p* < 0.001) and body mass index (B = -− 0.03; − 0.02 to − 0.04; *p* = 0.01) in the normal range. At last measurement, 11.7% of the total population had a HbA1c < 7.0% (53.0 mmol/mol).

**Conclusions:**

Across all measurements, coexisting T1D and CD is associated with lower HbA1c when compared to T1D alone. However, HbA1c is above target in both groups.

## Introduction

The association between type 1 diabetes and celiac disease (CD) is well documented, with the estimated prevalence of CD in people with type 1 diabetes varying worldwide. For example, in 57,375 people with type 1 diabetes from the Better Control in Pediatric and Adolescent Diabetes: Working to Create Centers of Reference (SWEET) registry, CD prevalence was 4.5%, with different prevalence among regions: from 1.9% in Asia/Middle East to 6.9% in Australia/New Zealand [1]. Elsewhere, recent data demonstrate a CD prevalence of 5.6% in children and adolescents with type 1 diabetes from Sweden [2], and in 52,721 people from the Prospective Diabetes Follow-up Registry (DPV) (Germany/Austria), the T1D Exchange Clinic Network (T1DX) (United States of America), the National Paediatric Diabetes Audit (NPDA) (United Kingdom [England/Wales]) and the Australasian Diabetes Data Network (ADDN) (Australia), the prevalence of CD in children and adolescents with type 1 diabetes was 3.2%, 1.9%, 3.8% and 7.7%, respectively [3]. Although most prevalence studies only include children and adolescents, figures are similar when extended to young adults (5.0%) [4], much higher than the pooled prevalence of 1.4% in the general population [5]; case detection being likely to reflect screening practices within the type 1 diabetes population and in at-risk cohorts.

Adverse effects of untreated CD include iron deficiency, anemia, abnormal cortical and trabecular bone, osteoporosis and growth retardation [6]. Some studies have demonstrated that the coexistence of type 1 diabetes and CD is associated with higher HbA1c, as well as an increased risk of hypoglycemia and microvascular complications [7–9]. Conversely, other studies have shown no difference or lower HbA1c and lower incidence of complications [3, 10–14]; a variation possibly occurring, in part, due to consideration around adherence to a gluten free diet in sample populations [14, 15].

There are limited data on HbA1c or therapy among adolescents and young adults with coexisting type 1 diabetes and CD [11, 12, 14]; this age range is important as it represents a distinct phase of maturation, and it is widely documented that in type 1 diabetes, HbA1c is typically higher in this population [16–18]. We hypothesized that HbA1c would be comparable among adolescents and young adults with type 1 diabetes, with or without CD.

## Methods

### Population

We used prospectively collected longitudinal data from the Australasian Diabetes Data Network (ADDN), a research collaboration among Australasian diabetes centers [19]. Inclusion criteria were type 1 diabetes (with or without CD), ≥ 1 HbA1c measurement, and at the last HbA1c measurement of age 16–25 years with diabetes duration ≥ 1 year; an age range chosen as it covers transition from paediatric to adult services, and because maturation is variably but generally completed by age 25 years [16]. Data extracted (June 1997 to March 2021) included socio-demographic variables such as sex, country of birth, self-identified Indigenous status and center, and clinical variables such as age at visit, CD diagnosis, date of visit, HbA1c, insulin regimen (twice daily injection (BD), multiple daily injection (MDI) or continuous subcutaneous insulin infusion (CSII)), total daily dosage (TDD) of insulin, blood pressure (BP) and body mass index (BMI).

### Definitions and approvals

BP was defined as being in hypertensive ranges when participants aged < 18 years had a systolic and/or diastolic BP at ≥ 95^th^ percentile and, for those aged ≥ 18 years, systolic BP ≥ 130 and/or diastolic BP ≥ 80 mmHg [20]. For those aged < 18 years, standardized BMI scores were based on Centers for Disease Control and Prevention reference data [21]. Overweight/obesity was defined as BMI standard deviation score (SDS) ≥ 85^th^ percentile for those aged < 18 years or BMI > 25 kg/m^2^ for those ≥ 18 years. All centers had Human Research or Health and Disability Ethics Committee approval for participation in ADDN, and the current analysis was approved by the University of the Sunshine Coast Human Research Ethics Committee, Australia (reference: E19116).

### Statistical methods

Descriptive statistics are reported as mean ± SD or number (%). HbA1c values were categorized into < or ≥ 7.0% (53.0 mmol/mol) based upon the International Society for Pediatric and Adolescent Diabetes (ISPAD) target of < 7.0% (< 53.0 mmol/mol) [22] and < or ≥ 9.0% (75.0 mmol/mol), a value widely considered to be a marker of very suboptimal glycemia. Results are stratified by presence or absence of CD, with differences between groups examined using Chi-square and t-tests. Generalized Estimated Equation (GEE) modelling was used for multivariable longitudinal analyses of HbA1c measurements across all visits. Explanatory variables included in the models were: type 1 diabetes duration, sex (male vs. female), presence or absence of CD, CSII (vs. BD/MDI) therapy, BP measurement in the hypertensive range (no/yes), and elevated BMI (no/yes); variables chosen based on clinical knowledge and previous literature. Goodness of fit was assessed using the two extensions of Akaike’s information criterion for model selection: quasi-likelihood under the independence model criterion (QIC) for choosing the best correlation structure and another QIC measure for choosing the best subset of predictors.

Results are reported as beta and 95% confidence intervals; *p* < 0.05 was considered significant and all assumptions were tested and met. Age at visit was not used in GEE models due to collinearity with diabetes duration. Analyses were performed using SPSS version 29™ software (IBM, New York).

## Results

### Last HbA1c measurement

A cohort of 6,480 adolescents and young adults with type 1 diabetes (52.6% male) met the inclusion criteria; data heralded from 24 centers across Australia and New Zealand (13 pediatric and 11 adult). Mean age was 18.3 ± 2.2 years, age at type 1 diabetes diagnosis was 9.1 ± 4.4 years, and diabetes duration was 8.7 ± 4.6 years. Place of birth was Australia or its territories in 85.1%, and New Zealand in 5.5%; 51 (1.2%) identified as Aboriginal and 66 (1.6%) as Māori. Most (52.2%) utilized MDI therapy, with 38.7% using CSII and 9.1% using BD injections; mean HbA1c was 8.8% ± 1.4 (72.9 ± 20.5 mmol/mol). In total, 362 (5.6%) adolescents and young adults had co-existing CD. Mean age at CD diagnosis was 10.2 ± 3.6 years, with CD diagnosed 2.0 ± 3.8 years after a type 1 diabetes diagnosis.

Characteristics of those with coexisting type 1 diabetes and CD vs. type 1 diabetes alone at last HbA1c measurement are shown in Table 1. Those with coexisting type 1 diabetes and CD were more likely to be female, younger, diagnosed with type 1 diabetes at a younger age and attend a pediatric center. Mean HbA1c did not significantly differ between those with coexisting type 1 diabetes and CD vs. type 1 diabetes alone; only 11.7% of the total population had a HbA1c < 7.0% (53.0 mmol/mol); 38.5% had HbA1c ≥ 9.0% (75.0 mmol/mol). The proportions that had a HbA1c measurement categorized as either < 7.0% (53.0 mmol/mol), 7.0–8.9% (53.0–75.0 mmol/mol) or ≥ 9.0% (75.0 mmol/mol) differed (*p* = 0.01), as did the proportions using BD, MDI or CSII therapy (*p* < 0.001). Adolescents and young adults with type 1 diabetes and coexisting CD had a trend towards higher TDD of insulin, less BP measurements in the hypertensive range and elevated BMI.

**Table 1:** Characteristics of people with type 1 diabetes in the ADDN registry at last HbA1c measurement

	Overall	CD		P value*
		Yes	No	
*n* =	6480	362	6118	
Male	3408 (52.6)	156 (43.1)	3252 (53.2)	< 0.001
Age (years)	18.3 ± 2.2	18.0 ± 2.0	18.3 ± 2.2	0.004
Age at type 1 diabetes diagnosis	9.1 ± 4.4	7.6 ± 4.4	9.2 ± 4.4	< 0.001
Type 1 diabetes duration (years)	8.7 ± 4.6	9.9 ± 4.5	8.6 ± 4.6	< 0.001
Pediatric center	5032 (77.7)	347 (95.9)	4685 (76.6)	< 0.001
HbA1c (%)	8.8 ± 1.4	8.7 ± 1.6	8.8 ± 1.9	0.11
HbA1c (mmol/mol)	72.9 ± 20.5	71.2 ± 17.7	73.0 ± 20.7	–
HbA1c categories				0.01
< 7.0% (53.0 mmol/mol)	760 (11.7)	36 (9.9)	724 (11.8)	–
7.0–8.9%	3222 (49.7)	208 (57.5)	3014 (49.3)	–
≥ 9.0% (75.0 mmol/mol)	2498 (38.5)	118 (32.6)	2380 (38.9)	–
Therapy:				< 0.001
BD	524 (9.1)	19.0 ± 5.7	505 (9.3)	–
MDI	3014 (52.2)	154 (46.2)	2860 (52.5)	–
CSII	2234 (38.7)	160 (48.0)	2072 (38.1)	–
TDD per kg (units)	1.4 ± 1.6	1.5 ± 4.2	1.4 ± 1.2	0.13
BP hypertensive range	1053 (35.2)	50 (29.1)	1003 (35.6)	0.08
Elevated BMI	1651 (39.6)	109 (40.1)	1542 (39.6)	0.87

*= *Comparing between CD presence or absence groups. Not all adolescents and young adults had all elements documented. BD* = *Twice-daily injections; BMI* = *Body mass index; BP* = *Blood pressure; CD* = *Celiac disease; CSII* = *Continuous Subcutaneous Insulin Infusion; MDI* = *Multiple daily injections; and TDD* = *Total Daily Dosage*

**Fig. 1:**
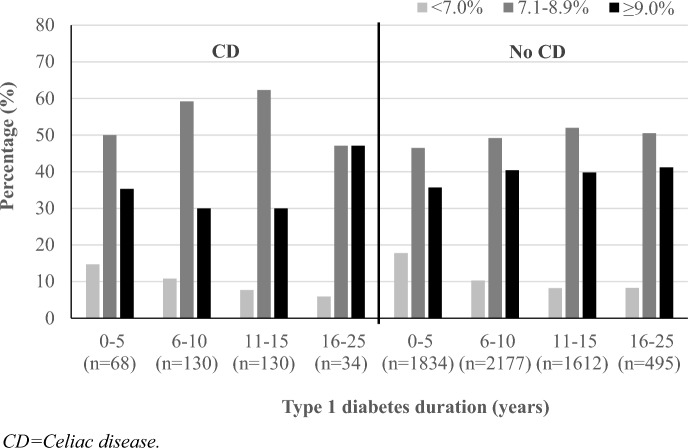
Values of HbA1c at last measurement stratified by type 1 diabetes duration and presence or absence of CD

Values of HbA1c at last measurement stratified by type 1 diabetes duration and presence or absence of CD are shown in Fig. 1; generally, as type 1 diabetes duration increased, the proportion of adolescents and young adults achieving an HbA1c < 7.0% (53.0 mmol/mol) decreased.

### All HbA1c measurements

Across all HbA1c measurements (n = 32,025) across all visits, there was a small but significant difference in the number of measurements in those with co-existing type 1 diabetes and CD vs. type 1 diabetes alone (5.5 ± 4.1 vs. 4.7 ± 3.8; *p* < 0.001). Mean HbA1c across all visits was 8.7 ± 1.8% (71.2 ± 20.0 mmol/mol) and was slightly lower in those with coexisting type 1 diabetes and CD (8.5 ± 1.5% (69.4 ± 16.8 mmol/mol) vs. 8.7 ± 1.8% (71.4 ± 19.8 mmol/mol); *p* < 0.001).

Using multivariable GEE modelling, lower HbA1c was associated with shorter type 1 diabetes duration, male sex, use of CSII therapy, coexistence of type 1 diabetes and CD, and BP and BMI in the normal range (Table 2). In subgroup analysis of those with coexisting diabetes and CD, lower HbA1c was associated with shorter type 1 diabetes duration (B = − 0.09, 95% CI = − 0.13 to − 0.04; *p* < 0.001), and use of CSII therapy (B = − 0.46, − 0.81 to − 0.12; *p* < 0.001).

**Table 2:** Variables associated with HbA1c in people with type 1 diabetes, with or without CD

Variable	Beta	95% CI	*P* value
Shorter type 1 diabetes duration	− 0.06	− 0.07 to − 0.05	< 0.001
Male sex	− 0.24	− 0.36 to − 0.11	< 0.001
CSII (vs. BD/MDI injection)	− 0.46	− 0.58 to − 0.34	< 0.001
Type 1 diabetes and CD (vs. alone)	− 0.28	− 0.48 to − 0.07	0.01
BP in normal (non-hypertensive) range	− 0.16	− 0.23 to − 0.09	< 0.001
BMI in normal range	− 0.03	− 0.02 to − 0.04	0.01

*n* = *7,691/32,025 visits. BD* = *Twice-daily injections; BMI* = *Body mass index; BP* = *Blood pressure; CD* = *Celiac disease; CSII* = *Continuous subcutaneous Insulin Infusion; and MDI* = *Multiple daily injections*

## Discussion

Our findings indicate that coexisting type 1 diabetes and CD is associated with lower HbA1c when compared to those with type 1 diabetes alone. Across all HbA1c measurements, those with coexisting type 1 diabetes and CD had a small but statistically significantly lower HbA1c; lower HbA1c was associated with shorter type 1 diabetes duration, male sex, use of CSII therapy, coexistence of type 1 diabetes and CD, and BP and BMI in the normal range. However, HbA1c remains above target in both groups. It is disappointing that, at last HbA1c measurement, only 11.7% of the total population had a HbA1c < 7.0% (53.0 mmol/mol), with 38.5% having a HbA1c ≥ 9.0% (75.0 mmol/mol).

Findings relating to glycemia in those with coexisting type 1 diabetes and CD are consistent with data in youth in the ADDN registry [17]. In our previous report, mean HbA1c was 8.8 ± 1.8% (72.2 ± 19.9 mmol/mol) and only 12.3% had an HbA1c < 7.0% (53 mmol/mol); lower HbA1c was associated with male sex and use of CSII therapy, with higher HbA1c associated with longer type 1 diabetes duration. However, when considering the impact of coexisting CD, findings differ to elsewhere in the literature. In youth with type 1 diabetes across three international registries, we have demonstrated that HbA1c was comparable [3].

ISPAD recommend screening for CD in young people at type 1 diabetes diagnosis and 1–2 years thereafter, with more frequent screening if clinically indicated or if there is a first-degree relative with CD [23, 24]. We previously reported a CD prevalence of 7.7% in youth, while only 5.1% of this older cohort had CD. It is recognized that CD is more common in those diagnosed with type 1 diabetes aged < 5 years [25], however it is also possible that screening for CD was conducted less frequently in the young adult population in ADDN [26]. The need for a more efficient, targeted diagnostic CD approach has been advocated. In a study undertaken in the Netherlands, for example, 42% of people with type 1 diabetes who came to develop CD were not diagnosed (with CD) until 10 years following type 1 diabetes onset [10]. Ideally, Australian national guidelines that were published almost a decade ago, should be revised and broadly implemented [27]. CD screening at type 1 diabetes diagnosis, within 2–5 years thereafter and at times where there are symptoms suggestive of CD, in addition to further research to determine screening frequency beyond 5 years of diabetes duration, has been advocated [25]. Various clinical indications for CD screening beyond just type 1 diabetes have also been shared [28]; especially important considering increasing worldwide CD incidence [29].

Our findings should be interpreted with some caution. We did not examine socioeconomic characteristics or input from differing healthcare professionals. Though our study did not consider dietetic input, there was a trend towards more visits in this population. Further, we did not consider adherence to a gluten free diet which, in youth with coexisting type 1 diabetes and CD, leads to regular growth and stable BMI, without any negative effect on HbA1c and insulin requirements [15]. We also did not examine loss to contact when transitioning from paediatric to adult type 1 diabetes healthcare services, and data were not available on use of use of continuous glucose monitoring in this population. ADDN data are also predominantly derived from public diabetes clinics; therefore data from general practice or private practice were not fully captured [30]. Finally, use of a tighter age range to represent adolescents and young adults, especially when considering typical ages of rebellion and discomfort, may have yielded differing results. Nevertheless, a major strength of our research is the sample size, and volume of available HbA1c measurements and clinical data; the size and scope of our database suggests our data are reasonably representative. Moreover, data were derived from across varying geographic regions.

In conclusion, we have demonstrated that coexisting type 1 diabetes and CD is associated with a small, but statistically significantly lower HbA1c, when compared to those with type 1 diabetes alone. However, HbA1c remains above target in both groups. Findings indicate that clinicians should be aware of risk factors for higher HbA1c, namely longer type 1 diabetes duration, BP in the hypertensive range and elevated BMI.
