# Correction to: Coexisiting type 1 diabetes and celiac disease is associated with lower Hba1c when compared to type 1 diabetes alone: data from the Australasian Diabetes Data Network (ADDN) registry

**DOI:** 10.1007/s00592-023-02160-6

**Published:** 2023-09-02

**Authors:** Steven James, Lin Perry, Julia Lowe, Kim C. Donaghue, Anna Pham-Short, Maria E. Craig, the ADDN Study Group, Geoff Ambler, Kym Anderson, Sof Andrikopoulos, Jenny Batch, Justin Brown, Fergus Cameron, Peter G. Colman, Louise Conwell, Andrew Cotterill, Jennifer Couper, Elizabeth Davis, Martin de Bock, Jan Fairchild, Gerry Fegan, Spiros Fourlanos, Sarah Glastras, Peter Goss, Leonie Gray, Peter Shane Hamblin, Paul Hofman, Dianne Jane Holmes-Walker, Tony Huynh, Sonia Isaacs, Craig Jefferies, Stephanie Johnson, Tim Jones, Jeff Kao, Bruce R. King, Antony Lafferty, Michelle Martin, Robert McCrossin, Kris Neville, Mark Pascoe, Ryan Paul, Alexia Peña, Liza Phillips, Darrell Price, Christine Rodda, David Simmons, Richard Sinnott, Carmel Smart, Monique Stone, Steve Stranks, Elaine Tham, Barbara Waddell, Glenn Ward, Ben Wheeler, Helen Woodhead, Anthony Zimmermann

**Affiliations:** 1 University of the Sunshine Coast, Moreton Bay Campus, 1 Moreton Parade, Petrie, 4502 Australia; 2 University of Melbourne, Parkville, Australia; 3 University of Western Sydney, Campbelltown, Australia; 4 University of Technology Sydney, Ultimo, Australia; 5 Prince of Wales Hospital, Randwick, Australia; 6 University of Toronto, Toronto, Canada; 7 Children’s Hospital at Westmead, Westmead, Australia; 8 University of Sydney, Camperdown, Australia; 9 University of New South Wales, Kensington, Australia

Correction to: Acta Diabetologica 10.1007/s00592-023-02113-z

‘Australasian Diabetes Data Network Study Group’ should be listed as named authors in the author group.

In the abstract, ‘coexistence of T1D and CD’ does not have a listed B value which is now updated as shown below

……coexistence of T1D and CD (B =  − 0.28; to − 0.48 to − 0.07……

Body mass index has a small negative value in both the abstract and Table 2, which is now corrected.

In **Australasian Diabetes Data Network (ADDN) Study Group members:** Jenny Batch should be Professor Jenny Batch. There is a ‘2’ incorrectly inserted in Prof Tony Huynh’s affiliation. This should say Queensland Children’s Hospital, Brisbane.

The original article has been corrected.

